# Creatinine-to-cystatin C ratio as muscle assessment tool and predictive value for mortality and sarcopenia in patients with chronic kidney disease: a meta-analysis

**DOI:** 10.3389/fnut.2025.1655488

**Published:** 2025-11-18

**Authors:** Wen-He Zheng, Yan-Ge Hu, Da-Xing Yu, Hui-Bin Huang

**Affiliations:** 1 Department of Critical Care Medicine, The Second People's Hospital Affiliated to Fujian University of Traditional Chinese Medicine, Fuzhou, China; 2 Department of Critical Care Medicine, Guang'anmen Hospital, China Academy of Chinese Medical Sciences, Beijing, China

**Keywords:** creatinine-to-cystatin C rate, chronic kidney disease, muscle mass, mortality, meta-analysis

## Abstract

**Background:**

The creatinine-to-cystatin C ratio (CCR) has been developed as a novel biomarker of sarcopenia and prognostic evaluation in various hospitalized populations. However, evidence supporting the use of CCR in patients with chronic kidney disease (CKD) remains limited. Thus, we aimed to evaluate whether CCR could be a marker of muscle mass for predicting prognosis in patients with CKD.

**Methods:**

We searched PubMed, Embase, Wanfang, China National Knowledge Infrastructure, Web of Science, and Cochrane Library databases up to March 15, 2025. Studies were included if they reported a relationship between CCR and muscle measurements or prognosis in adults with CKD. The risk of bias in non-randomized studies-of exposures tool was used to assess the quality of the study. The primary outcome was all-cause mortality. This review was conducted in accordance with the Preferred Reporting Items for Systematic Reviews and Meta-Analysis guidelines.

**Results:**

Nine studies (seven cohort and two cross-sectional studies) involving 31,673 adults were included. The quality of the included studies ranged from moderate to high. Pooling the results from multifactorial analyses showed that CCR can reliably predict mortality, either using CCR as a category variable [*n* = 24,778; hazard ratio (HR) = 2.16; 95% CI, 1.40–2.88; *I*^2^ = 48%] or a continuous variable (*n* = 3,313; HR = 0.73; 95% CI, 0.57–0.93; *I*^2^ = 68%). CCR was positively correlated with handgrip strength (*n* = 874; *r* = 0.38, *P* < 0.001) and skeletal muscle index (*n* = 357; *r* = 0.42, *P* < 0.001). Similarly, the area under curves (AUC) suggested that CCR had poor-to-fair diagnostic efficacy for handgrip strength (AUC = 0.640; 95% CI: 0.605–0.0.675), skeletal muscle index (AUC = 0.684; 95% CI: 0.596–0.772), and sarcopenia (AUC = 0.720; 95% CI: 0.619–0.822). For nutrition status, lower CCR was associated with significantly lower albumin but not body mass index.

**Conclusions:**

This meta-analysis suggests that CCR could serve as a valuable tool for evaluating muscle mass, as well as an indicator of nutritional status and an independent predictor of prognosis in patients with CKD. These findings encourage the use of CCR in this patient population. However, more high-quality studies are needed to confirm these findings.

**Systematic review registration:**

https://inplasy.com/inplasy-2022-9-0097/, identifier: NPLASY202290097.

## Introduction

Chronic kidney disease (CKD) is an increasingly prevalent global health issue, significantly raising risks of morbidity and mortality (1, 2). Patients with CKD experience accelerated muscle protein breakdown due to uremia, which dietary interventions often fail to interrupt this process, leading to progressive muscle waste (3, 4). This muscle loss frequently manifests as sarcopenia, a condition characterized by the progressive and simultaneous decline of muscle mass, strength, and function, which is a primary contributor to physical disability, frailty, and elevated mortality in this population (5–7).

A recent meta-analysis confirmed that diagnosed sarcopenia is also associated with significantly increased mortality risk in dialysis patients (HR:1.87; 95% CI: 1.35–2.59) (5). The high prevalence of sarcopenia in CKD is driven by a confluence of factors unique to this population, including chronic inflammation, metabolic acidosis, malnutrition and hormonal imbalances, which collectively create a catabolic milieu that exacerbates the loss of both muscle mass and quality (8). Notably, decreased muscle strength occurs more frequently in patients with CKD than in healthy individuals, often directly correlating with the stages of CKD (9, 10). Therefore, early identification of muscle loss is critical for improving outcomes in these patients.

The accurate assessment of sarcopenia in CKD remains challenging. While imaging techniques like dual-energy X-ray absorptiometry (DXA) are considered standard for measuring muscle mass, their accuracy can be compromised by fluid overload, a common complication in CKD (11). More precise methods like magnetic resonance imaging, computed tomography (CT), are not routinely feasible for screening, and bioimpedance analysis requires careful interpretation in the context of hydration status (12, 13). Consequently, there is a pressing need for accessible and reliable serum biomarkers to identify patients at risk.

The creatinine-to-cystatin C ratio (CCR) has emerged as a promising tool in this regard. Serum creatinine, derived from muscle metabolism, and cystatin C, produced ubiquitously and unaffected by muscle mass, are both filtration markers used to estimate renal function. Their rate effectively isolates the muscle-derived component of creatinine from the glomerular filtration rate (GFR), thereby serving as a surrogate indicator for skeletal muscle mass (14). In 2016, Kashani et al. (14) formally proposed CCR as a “sarcopenia index” correlated with muscle mass measured by CT. Subsequent research, synthesized in our 2022 meta-analysis of 38 observational studies, confirmed that CCR is a robust predictor of mortality and adverse outcomes across diverse clinical settings (15).

However, a critical evidence gap remains. The vast majority of studies validating CCR, including those in our previous meta-analysis, systematically excluded patients with severe renal impairment. Therefore, it is unknown whether the established cut-offs for sarcopenia remain valid or if CCR retains its discriminatory power in the context of impaired renal function.

Several recent publications have been published in this field (16–18). Therefore, we aimed to conduct a systematic review and meta-analysis to evaluate the relationship between CCR, muscle mass, and clinical outcomes in the CKD population, thereby providing essential evidence on its utility in this specific clinical context.

## Methods

This study was reported according to the recommendations of the preferred reporting items for systematic reviews and meta-analyses checklist (19) (Supplementary File 1) and registered at the international platform of registered systematic review and meta-analysis protocols (NPLASY202290097).

### Search strategy

We conducted a literature search in the following databases: PubMed, Embase, Cochrane Library, Web of Science, Wanfang, and China National Knowledge Infrastructure, covering the earliest available year in each database up to March 15, 2025. The search strategy was designed by H-BH and executed by W-HZ and Y-GH. We used Boolean operations to combine keywords and related medical subject headings as search terms for “sarcopenia,” “muscle atrophy,” and “chronic kidney disease” with no language restrictions. The details of the search strategy are described in Supplementary File 2. The gray literature was searched at https://scholar.google.com/, ClinicalTrials.gov, and https://www.base search.net/. Additionally, we checked the reference lists of the associated articles, reviews, and meta-analyses to identify potentially relevant studies.

### Inclusion and exclusion criteria

We defined eligibility criteria using the PICOS principle. Participants: adult patients with a confirmed diagnosis of CKD, with or without dialysis. Intervention: muscle atrophy identified through CCR. Comparators: adult patients diagnosed with CKD and without muscle atrophy. Outcomes: to explore whether CCR could be a marker of muscle mass and assess the association between CCR and all-cause mortality, as well as other clinical outcomes. Design: included cohorts, case-control studies, or randomized controlled trials.

Exclusion criteria consisted of studies involving patients with acute kidney injury, children, or pregnant women, as well as study designs such as reviews, case reports, conference abstracts, expert opinions, or animal studies.

### Study outcomes

The primary outcome was the predictive value of CCR for mortality in patients with CKD at the longest follow-up available. Secondary outcomes included associations between CCR and CT-based muscle measurements (muscle area, strength, or function) and nutrition status (body mass index and albumin levels).

### Literature selection and data extraction

We used EndNote reference management software to screen the literature. Two authors (Y-GH and J-HS) independently extracted the following data from the final studies included: the first author, year of publication, sample size, patient characteristics [sex, age, and body mass index (BMI)], CCR, muscle measurements, follow-up duration, and predefined outcomes. Any disagreements were resolved by a third author (H-BH).

### Quality assessment of literature

Cohen's kappa (κ) value was calculated to estimate the extent of concordance between the two authors who conducted a literature search and checked eligibility and data extraction. The quality of each included study was evaluated using the Risk Of Bias In Non-randomized Studies–of Exposures (ROBINS-E) tool (20). We used the grading of recommendations, assessment, development and evaluation (GRADE) tool to rigorously assess the quality of evidence for the primary outcomes (21). This assessment focused on five critical parameters: risk of bias, inconsistencies between studies (heterogeneity), indirectness, imprecision (the risk of random error), and publication bias. Each parameter was clearly rated as high, moderate, low, or very low, ensuring a comprehensive evaluation of the evidence.

### Statistical analysis

A meta-analysis was conducted to evaluate the data reporting formats identified in the included studies. For studies that reported outcomes from regression analyses, we extracted the 95% confidence intervals (CIs) for hazard ratios (HRs) and pooled them using the inverse variance method. Both univariate and multivariate regression results were combined to assess the reliability of the findings. In studies that provided correlation data, we performed a meta-analysis to calculate the pooled correlation coefficient (*r*) using established methods. For studies presenting comparative data between different groups, we evaluated the relationships by pooling the odds ratios (ORs) from these groups.

To assess heterogeneity, we applied the *I*^2^ statistic, categorizing the results as low (*I*^2^ < 50%) or high (*I*^2^ >50%) (22). When significant heterogeneity was detected, we used a random-effects model; otherwise, a fixed-effects model was employed. We assessed publication bias by visually exploring funnel plots for asymmetry when at least 10 studies were included in the analysis. Additionally, sensitivity analyses were performed by removing individual studies one at a time to determine their impact on the overall effect estimate. All statistical analyses for this meta-analysis were conducted using R software, and a *P*-value of < 0.05 was considered statistically significant.

## Results

### Trial identification and study characteristics

**Figure 1:**
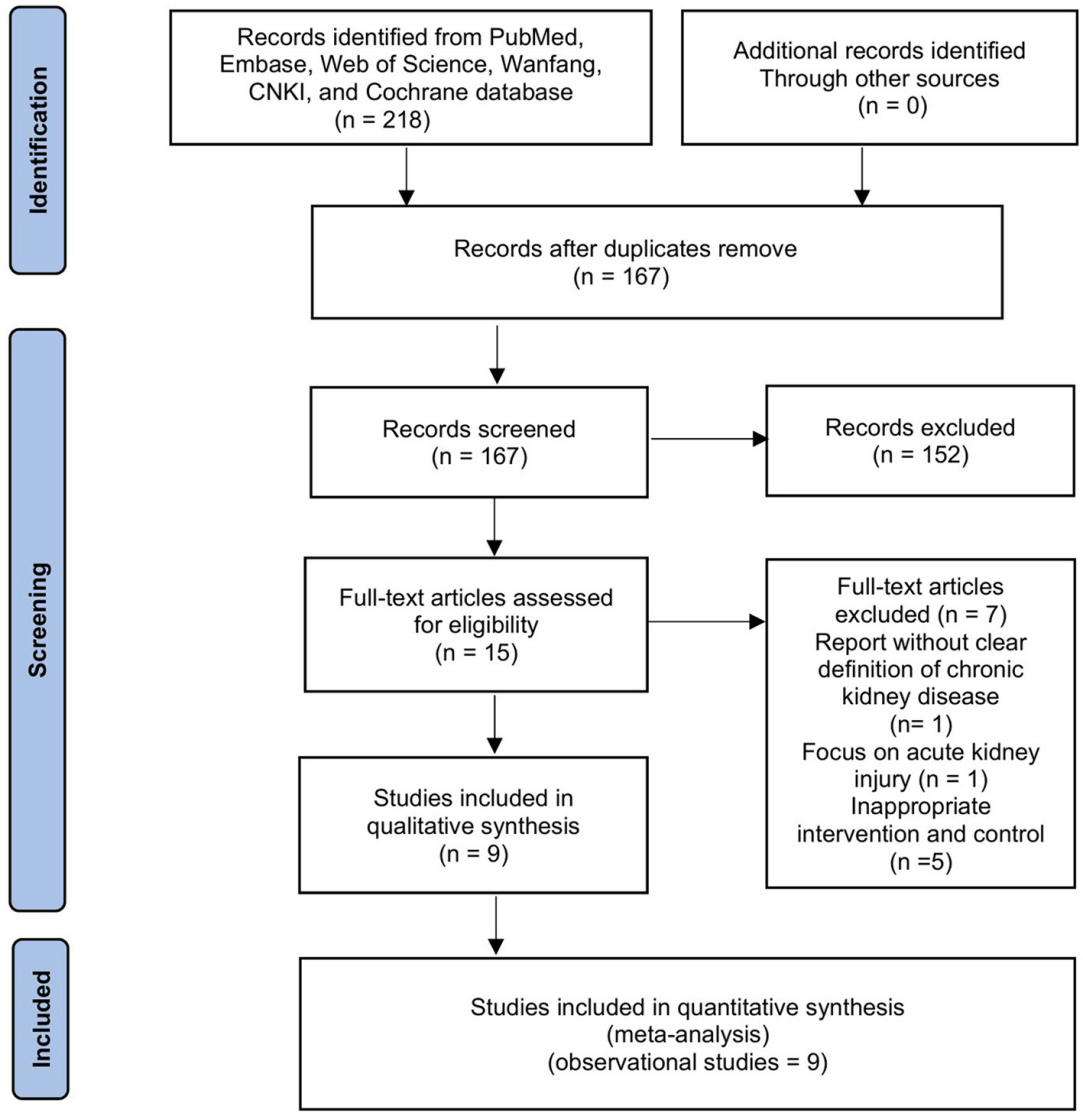
Selection process for studies included in the meta-analysis.

The initial search resulted in 218 entries. After removing duplicates and screening titles and abstracts, 16 articles were selected for full-text review. Two authors independently assessed these articles, and seven studies were excluded (report without a clear definition of CKD, *n* = 1; focus on acute kidney injury, *n* = 1; inappropriate intervention and control, *n* = 5). Thus, nine cohort studies involving 31,673 patients met our inclusion criteria (Figure 1) (16–18, 23–28). In particular, in the two studies that recruited mixed populations, only adults with CKD were included in our final meta-analyses.

Table 1 summarizes the main characteristics of the included studies. Data related to the treatment of patients with CKD are summarized in Supplementary File 3. These studies were published between 2019 and 2023 in China (*n* = 4) (17, 26–28), the USA (*n* = 1) (16), and Korea (*n* = 4) (18, 23–25). The mean age of the participants was as follows: 50–60 years in two studies (25, 28), 60–70 years in four studies (16, 18, 23, 27), 70–80 years in two studies (24, 26), and unavailable in one study (17). The mean BMI ranged from 22.4 to 26.1 kg/m^2^. Seven studies focused on non-dialysis CKD patients, whereas the remaining recruited hemodialysis CKD patients (18, 28). Six studies examined the effect of CCR on mortality using regression analyses (16, 17, 24–26, 28), and two studies examined the mortality between low or high CCR groups (25, 28). Regarding secondary outcomes, three studies evaluated the effect of CCR on muscle measurements of hand grip strength (HGS) and skeletal muscle index (SMI) (18, 23, 27).

**Table 1:** Characteristics of included studies in the current meta-analysis.

**Study**	**Country**	**Design**	**Study period**	**Population**	**Sample size**	**Mean age, year**	**Male,%**	**BMI**,	**Mean eGFR, ml/min/1.73m^2^**	**Baseline CCR**	**Predefined outcomes**
Hyun (25)	Korea	P, MC	2011–2016	CKD, ND	1,966	53.6	61	24.57	53.6	NA	①
Hwang (24)	Korea	R, SC	2003–2020	CKD, ND	4,301	70.1	43	25.6	27.4	1.3	①
Lin (26)	China	CS, SC	2015.4–2016.2	CKD, ND	272	66.5	57	26.1	36.5	1.00	③②
Rizk (16)	USA	R, MC	2004.10–2019.9	CKD, ND	22,316	67	95	NA	< 60	NA	①
Yajima (18)	Korea	CS, SC	2020.1–2020.12	CKD, D	85	67.8	68.2	22.4	1.5	1.5	③②④
An (23)	Korea	R, SC	2015.3–2020.2	CKD, ND	517	67	66.2	25.5	40.0	NA	③④
Shi (17)	China	R, MC	1999–2019.12	CKD, ND	851	NA	NA	NA	< 60	1.17	①
Zhang (28)	China	R, SC	2014.1–2019.12	CKD, D	224	50.5	44.2	20.5	NA	1.85	①
Lin (27)	China	R, SC	2016.2–2018.12	CKD, ND	1,141	71	58.2	25.5	32	0.98	①

R, retrospective; BMI, body mass index; CCR, creatinine/cystatin C ratio; CKD, chronic kidney disease; CS, cross-sectional; C, D, dependence; multi-centers; eGFR, estimated glomerular filtration rate; NA, not available; ND, not dependence; P, prospective; SC, single-center.

① Mortality; ② Skeletal muscle index; ③ Hand grip strength; ④ Sarcopenia.

### Study quality

Evaluation of the nine studies using the ROBIN-E tool revealed a range related to the overall risk of bias, including low risk (*n* = 4), some concerns (*n* = 4), and high risk (*n* = 1). Most studies have been judged poorly due to incomplete analysis of the two domains, bias due to confounding and measurement of the outcomes (Supplementary File 4). Regarding the eligibility of relevant and irrelevant articles from our study, authors were almost unanimous (Cohen's κ = 0.96; % agreement 97.63). The GRADE quality of evidence evaluation for mortality is outlined in Supplementary File 5.

### Prediction of mortality by CCR

**Figure 2:**
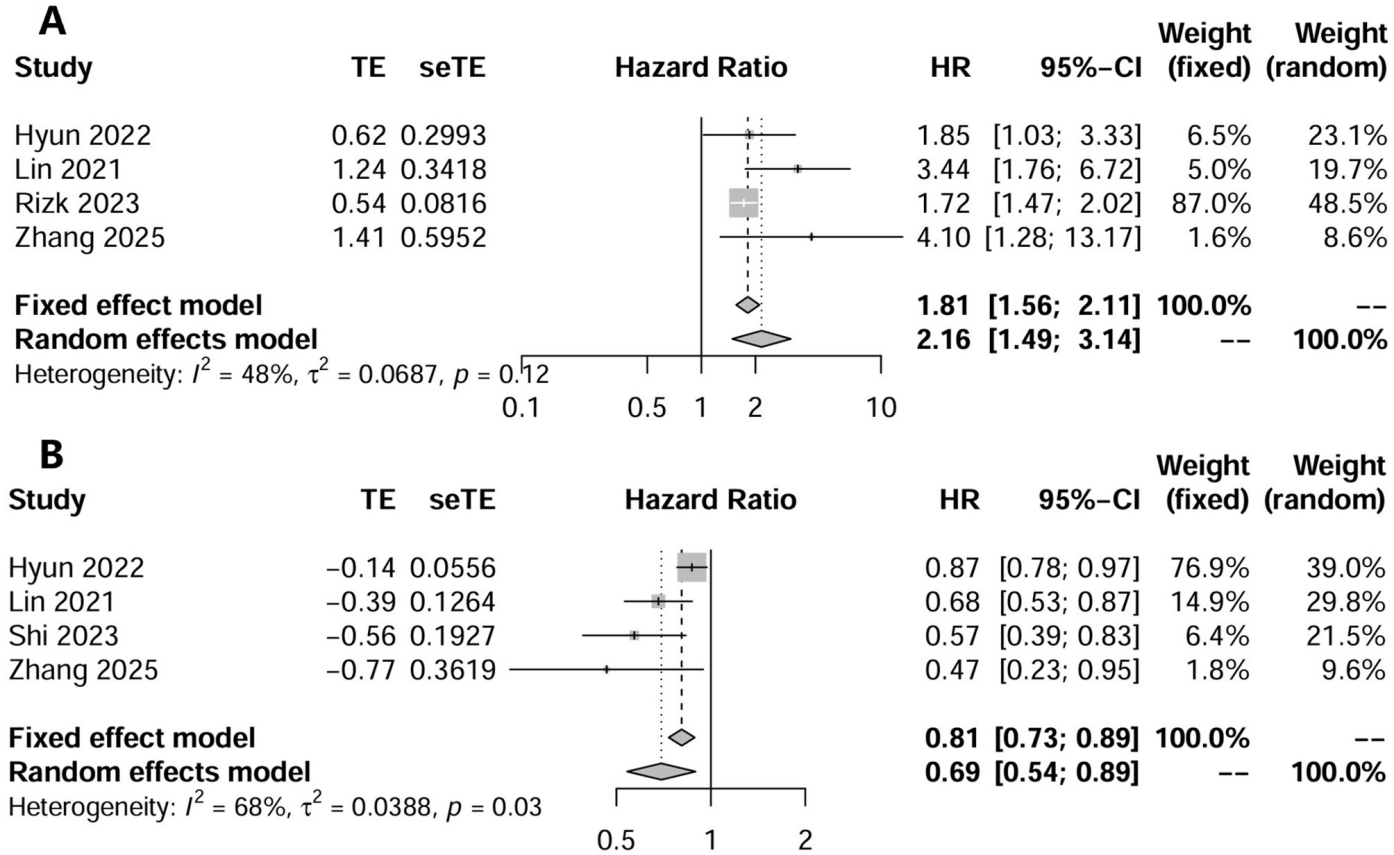
The forest plot in assessing the impact of creatinine/cystatin C ratio as a category variable on mortality by unadjusted regression analysis **(A)**; and adjusted regression analysis **(B)**.

**Figure 3:**
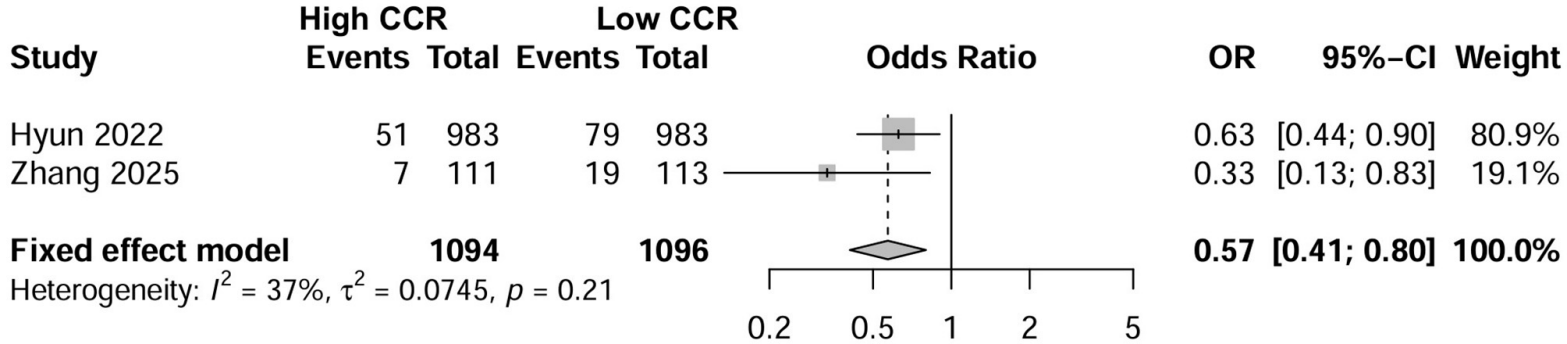
Forest plot showing the effects of high vs. low levels of creatinine-to-cystatin C ratio on mortality.

Six studies investigated the effect of CCR on all-cause mortality in CKD patients using multifactorial HR analysis (16, 17, 24–26, 28). Four studies reported risk assessments based on the lowest CCR quartile (16, 25, 26, 28). Pooling results revealed that a low CCR was significantly associated with mortality (HR = 2.16; 95% CI, 1.49–3.14; *I*^2^ = 48%; Figure 2A). Four studies treated CCR as a continuous variable, and the pooled results showed that a higher CCR was associated with a lower risk of mortality (HR = 0.69; 95% CI, 0.54–0.89; *I*^2^ = 68%; Figure 2B) (17, 25, 26, 28). Two studies compared the mortality risk between the high and low CCR groups and showed that CKD patients with a high CCR had significantly lower mortality (OR = 0.57; 95% CI, 0.41–0.80; *I*^2^ = 37%) than those with a low CCR (25, 28) (Figure 3). Subsequent sensitivity analyses by excluding a study on a case-by-case basis did not significantly change the combined results. We conducted *post-hoc* meta-regression analyses for dialysis status, eGFR, and age. The results of these regressions indicated that these factors did not account for the heterogeneity observed in the combined results (Supplementary File 6). In addition, one study showed that CCR was a fair predictor of mortality [area under the curve (AUC) = 0.652, 95% CI, 0.552, 0.753, *P* = 0.003].

Two studies conducted subgroup analyses that explored the effects of age and sex on mortality. In the study by Lin et al., the authors found that subgroup analyses based on male (HR = 0.71; 95% CI, 0.521–0.96), female (HR = 0.65; 95% CI, 0.43–0.98), and older age (>65 years; HR = 0.64; 95% CI, 0.48–0.84) supported the prognostic improvement of high CCR. However, Hyun et al. found that only patients in the older age group (>50 years; HR = 0.36; 95% CI, 0.19–0.68) benefited from high CCR.

### The relationship between CCR and muscle measurements

**Figure 4:**
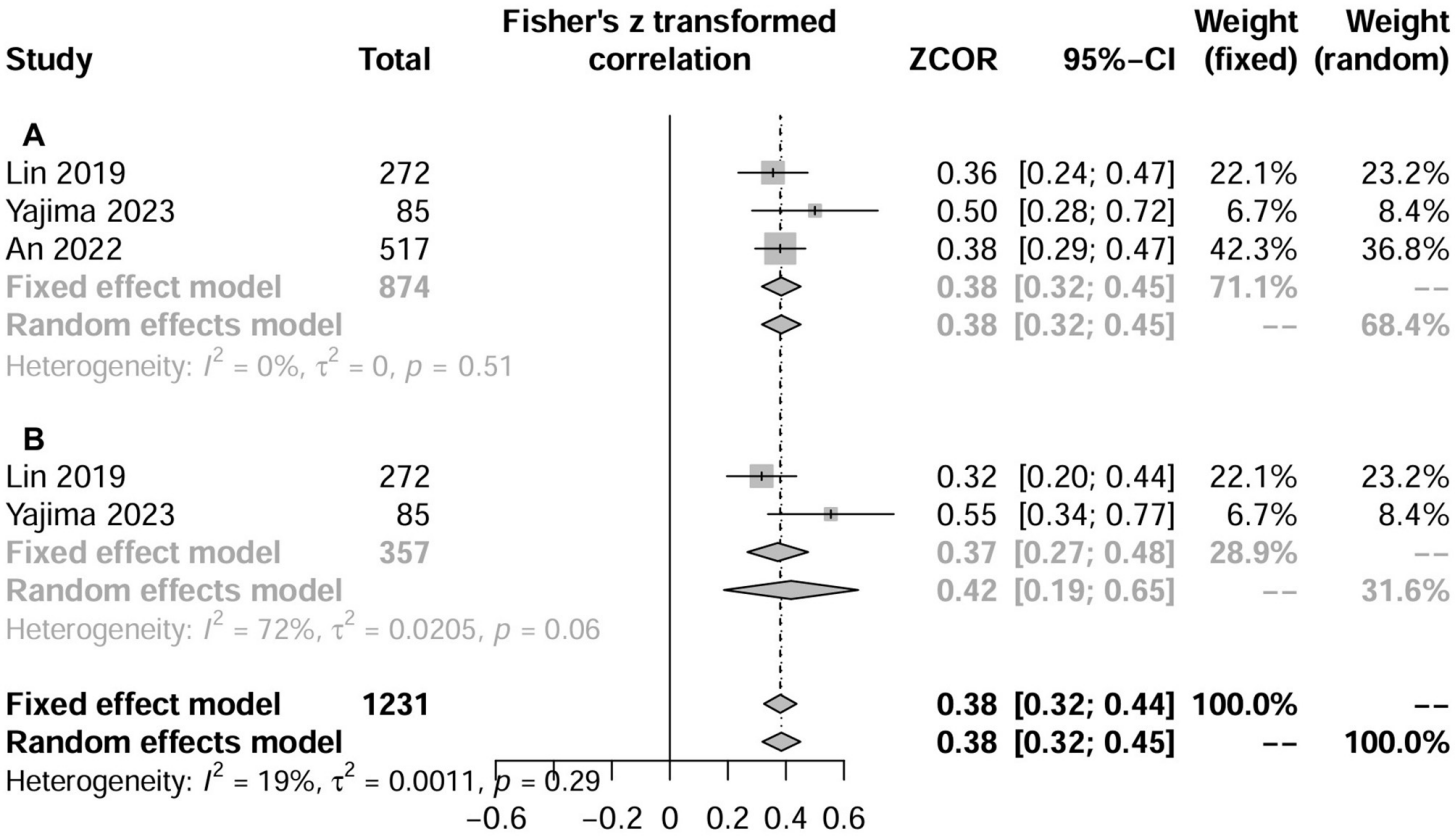
Forest plot showing the relationship between serum creatinine/cystatin C ratio and handgrip strength **(A)** or skeletal muscle index **(B)** using the correlation coefficient. HGS, hand grip strength; SMI, skeletal muscle index.

Three studies (*N* = 2,755) evaluated the correlation between CCR and muscle measurements using the correlation coefficient (18, 23, 27). The pooled results showed that CCR was positively correlated with both HGS (three studies, *r* = 0.38, 95% CI, 0.19–0.65; *P* < 0.001; Figure 4A) and SMI (two studies, *r* = 0.421, 95% CI, 0.32–0.45; *P* < 0.001; Figure 4B). Similarly, three studies (*N* = 1,208) showed that CCR was a fair predictor of HGS (three studies, AUC = 0.640; 95% CI: 0.605–0.0.675) (18, 23, 27), SMI (three studies, AUC = 0.684; 95% CI: 0.596–0.772) (18, 27), and sarcopenia (two studies, AUC = 0.720; 95% CI: 0.619–0.822) (18, 23).

In addition, An et al. (23) suggested that patients with sarcopenia had significantly lower CCR than patients without sarcopenia; this result was mostly further confirmed when patients were classified according to age, sex, eGFR, and BMI. Lin et al. (27) found that CCR was an independent risk factor for sarcopenia, low lean tissue index and low HGS risk, all adjusted ORs between 2.59 and 7.18, with *P* values ranging from 0.048 to < 0.0001.

### The relationship between CCR and nutrition status

**Figure 5:**
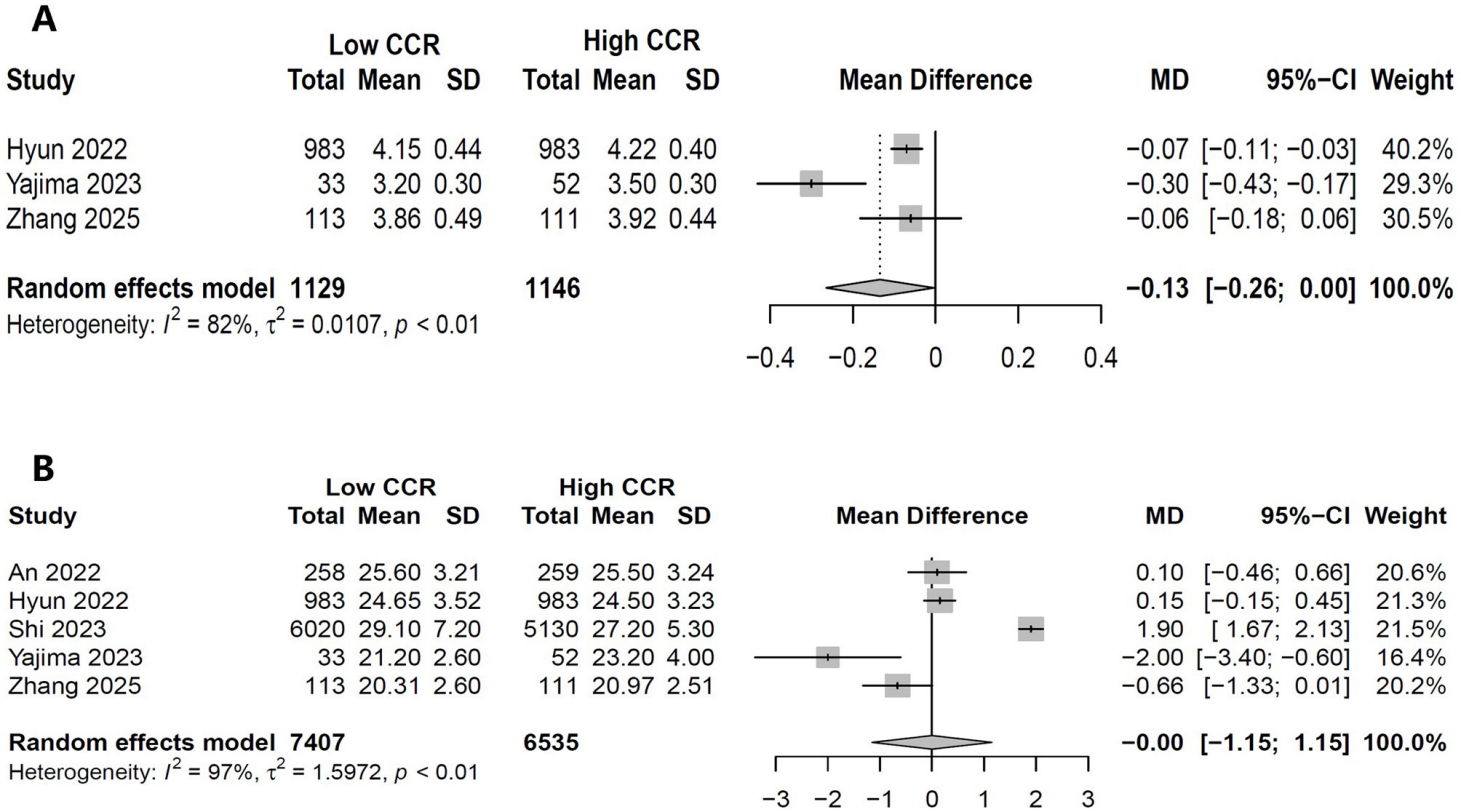
Forest plot showing the effects of high vs. low levels of creatinine-to-cystatin C ratio on serum albumin **(A)** and body mass index **(B)**.

Three studies (*N* = 2,275) compared albumin levels between high and low CCR (18, 25, 28). The pooled results showed that compared with high CCR, low CCR had significant lower albumin (MD, −0.134 g/dl, 95% CI, −0.2649 to −0.0395; *P* = 0.043; Figure 5A). Five studies (*N* = 13,942) compared BMI between high and low CCR and showed no difference between groups (MD, −0.0006 kg/m^2^, 95% CI, −1.15 to 1.15; *P* = 0.9992; Figure 5B).

## Discussion

In this meta-analysis, we included nine studies involving 31,673 patients to evaluate the value of CCR in patients with CKD. Our results indicate that muscle loss occurs frequently among CKD patients. CCR can reliably predict mortality rates in CKD patients, a conclusion supported by regression analysis and analyses of different CCR levels. Furthermore, The CCR shows a significant correlation with muscle indicators such as SMI and HGS, suggesting that CCR is an ideal alternative biomarker for diagnosing sarcopenia in this population. Limited evidence suggested that CCR was a fair predictor of HGS, SMI, and sarcopenia. Additionally, lower CCR had significantly lower serum albumin levels but not BMI.

### Clinical application of CCR

The CCR serves as a surrogate marker of muscle mass by leveraging the distinct origins of its components: creatinine, derived from muscle metabolism, and cystatin C, produced by all nucleated cells. In stable renal function, a lower CCR primarily indicates reduced muscle mass. However, in advanced CKD, its interpretation becomes more complex (29, 30). As highlighted by Sonne et al. (2020), a significant depletion of the muscle creatine pool occurs, which is the direct precursor for creatinine (31). Therefore, a low CCR in advanced CKD may reflect not only a loss of muscle mass but also a state of intramuscular creatine depletion, further compromising creatinine generation and amplifying the ratio's decline.

In 2016, Kashini et al. (14) first validated the correlation between muscle mass and CCR in critically ill patients. Since then, additional studies have affirmed the relationship between CCR and muscle measurements across various populations (32–34), highlighting its growing clinical importance. It provides a straightforward method for assessing muscle mass in hospitalized patients at high metabolic risk.

A meta-analysis published in 2022 suggested that CCR can serve as a surrogate for nutritional screening and muscle measurements tools (15). Moreover, CCR has been found to significantly correlate with clinical outcomes in ICU patients (15). However, it is important to note that most CCR studies have excluded patients with CKD or AKI due to concerns about the stability of individuals with renal insufficiency. Therefore, we conducted this meta-analysis to investigate this issue.

### Comparison with previous studies

Our study is the first to evaluate the clinical significance of CCR in patients with CKD, particularly its role as a reliable biomarker for sarcopenia and in assessing clinical prognosis. Our findings align with the previous meta-analyses evaluating sarcopenia in CKD patients, including studies utilizing ultrasound and the CT-assessed SMI (5, 35), all of which highlight the poor clinical outcomes associated with sarcopenia. Therefore, our study adds a new population to the evidence for the systematic review of novel biomarkers for sarcopenia.

Furthermore, we comprehensively assessed the prognostic value of CCR in CKD patients, focusing on mortality differences across varying CCR levels and the linear relationship between CCR and mortality. In the later analysis, we pooled adjusted data from each included study (16, 17, 25, 26, 28), minimizing the influence of clinical confounders. Our sample size comprised more than 31,000 participants, providing robust statistical power for sensitivity analyses based on various potentially influential factors. The results remained consistent, further reinforcing the reliability of our main findings.

### Interpreting our results

This study presents new biomarker evidence linking sarcopenia to poor prognosis in patients with CKD. Low CCR levels indicate muscle loss, which has been shown to influence outcomes across various patient populations (5). The molecular mechanisms behind sarcopenia in CKD are complex and closely related to multiple factors, including inflammation, oxidative stress, insulin resistance, vitamin D deficiency, intestinal dysbiosis, anorexia, hemodialysis, metabolic acidosis, and uremic toxins (3, 36). These factors activate downstream protein hydrolases, which lead to increased muscle proteolysis and reduced protein synthesis, ultimately resulting in skeletal muscle atrophy. Patients on dialysis often exhibit lower muscle strength and more severe sarcopenia compared to healthy adults. Moreover, cardiovascular disease remains the leading cause of death among individuals with CKD (25, 37). Sarcopenia significantly contributes to increased mortality, both cardiovascular and non-cardiovascular, in this population (25, 38).

Several clinical considerations clarify the predictive value of CCR as a marker of muscle mass in assessing prognosis for patients with CKD: (1) Beyond accelerated proteolysis, many CKD patients experience inadequate dietary energy and protein intake, thus negatively impacting their prognosis. (2) Muscle atrophy can lead to limited mobility, loss of independence, and increased vulnerability to disease complications. (3) Impaired muscle function and delayed rehabilitation in CKD patients elevate their risk of poor outcomes (18, 23, 26, 27). (4) Meta-analyses have shown that individuals with low muscle mass have a higher prevalence of chronic diseases and cardiovascular complications, longer hospital stays, and greater rates of disease recurrence and readmission, ultimately contributing to increased mortality (5, 15). (5) Sarcopenia indicates poorer nutritional status and is associated with complications from hospital-acquired infections. For example, research by Zhang et al. (39) found that CCR predicts a higher likelihood of septic shock in patients. In conclusion, CCR reflects that muscle wasting is a critical factor influencing negative outcomes in patients with CKD.

The pooled results of this study indicated that the CCR was significantly associated with ASMI (*r* = 0.38) and HGS (*r* = 0.38) in patients with CKD. These findings align with previous meta-analyses involving hospitalized patients, which reported correlations of 0.37 and 0.35, respectively (15). Additionally, a recent meta-analysis of hospitalized patients demonstrated that serum CCR has moderate diagnostic accuracy for sarcopenia, with a summary receiver operating characteristic AUC of 0.78 (95% CI: 0.74–0.81) (40). In our study, two of the included studies reported AUC values of 0.805 (18) and 0.605 (23) for the accuracy of CCR in diagnosing sarcopenia among CKD patients. Thus, CCR in CKD patients meets the current guideline requirements for the definition of sarcopenia (6). Similar to other non-CKD populations (such as critically ill, oncologic, and surgical patients), CCR may also serve as a reliable biomarker for screening sarcopenia in CKD patients. However, it is noteworthy that only two of the included studies focused on dialysis-dependent CKD patients (18, 28), and only one study reported that CCR demonstrated excellent predictive ability for muscle mass, strength, and sarcopenia (18). Therefore, more data on dialysis-dependent CKD patients are required to validate our findings.

This meta-analysis examined CKD patients with or without dialysis dependencies. Serum creatinine and cystatin C concentrations are periodic blood tests already performed in this population. Consequently, sarcopenia can be detected early using a simple, easy, and inexpensive biomarker of CCR, allowing for better medical management strategies and treatment goals.

Incorporating CCR into risk assessments for CKD patients can enhance our understanding of a patient's capacity for muscle mass, strength, and function. This strategy can help guide treatment decisions more effectively. For CKD patients with sarcopenia, regularly monitoring CCR, along with providing nutritional support and exercise rehabilitation programs (41, 42), can reduce complications and improve overall prognosis, ultimately enhancing activity levels throughout their lives.

### Limitations

Our meta-analysis has several limitations. (1) The observational design of all included studies prevents any causal inferences. Additionally, the retrospective nature of the studies, which included only patients who underwent CCR testing, may have resulted in selection bias. (2) Few studies used validated sarcopenia definitions. Moreover, some outcomes, particularly those measuring muscle parameters such as ASMI and HGS, were included in only a limited number of analyses, so their results should be interpreted cautiously. (3) The uneven distribution of underlying diseases, duration of CKD, proteinuria, therapeutic drugs, and dietary profiles among the CKD patients in these studies may have different prognostic implications. In addition, there are different definitions of sarcopenia/muscle outcomes (DXA, BIA, CT, HGS) and lack of standardized CCR cut-off values, which may affect the accuracy of the findings. (4) Cystatin C is a protease inhibitor, and its levels can be elevated in some non-renal, non-muscle conditions such as hyperthyroidism, obesity, metabolic syndrome, type II diabetes mellitus, and various inflammatory conditions. In addition, residual confounding by inflammation (cystatin C is up-regulated by IL-6), lack of longitudinal change in CCR, and the possibility of reverse causality might potentially affect our results. (6) Only a small number of studies that reference to the impact of CCR on nutrition, and more research is needed to clarify the predictive value of CCR compared to existing tools such as the malnutrition inflammation indicator and the geriatric nutritional risk index (43). Finally, most studies were conducted in Asian countries, indicating a need for more data from other ethnic groups to validate these findings.

## Conclusion

Our results revealed significant correlations between CCR and the muscle mass and quality evaluation in CKD patients. Higher CCR levels are associated with a lower risk of all-cause mortality in this patient population. Therefore, CCR could be a novel prognostic biomarker for CKD patients, providing better information for decision-making related to muscle mass and quality management and predicting survival and other important clinical outcomes.

## Data Availability

The original contributions presented in the study are included in the article/Supplementary material, further inquiries can be directed to the corresponding author.
