# Supplementary material for: Creatinine-to-cystatin C ratio as muscle assessment tool and predictive value for mortality and sarcopenia in patients with chronic kidney disease: a meta-analysis
Source: Front Nutr. 2025 Nov 18;12:1655488. doi: 10.3389/fnut.2025.1655488 (PMC12669093; doi:10.3389/fnut.2025.1655488)
Supplement: Supplementary file 1 [file Data_Sheet_1.DOCX]

**Creatinine/ cystatin C ratio as muscle assessment tool and predictive value of prognosis in patients with chronic kidney disease: a meta-analysis**

**Supplementary material**

**Additional file 1:** Checklist following PRISMA guideline ……………………………………………………………………...………1

**Additional file 2:** Search Strategy…………………………………………………………………………………..……………………...……..5

**Additional file 3:** Data related to the characteristics of patients with CKD………………………………….…...................8

**Additional file 4:** Quality assessment and overall risk of bias of included studies………………….……………….......10

**Additional file 5:** GRADE evaluation…………………………….…………………………………………………..…………………….......11

**Additional file 6:** Post-hoc meta-regression analyses of the primary outcome……………………………………….…..12

**Additional file 1: PRISMA checklist**

| **Section/topic** | **#** | **Checklist item** | **Reported on page #** |
| --- | --- | --- | --- |
| **TITLE** | | |  |
| Title | 1 | Identify the report as a systematic review, meta-analysis, or both. | 1 |
| **ABSTRACT** | | |  |
| Structured summary | 2 | Provide a structured summary including, as applicable: background; objectives; data sources; study eligibility criteria, participants, and interventions; study appraisal and synthesis methods; results; limitations; conclusions and implications of key findings; systematic review registration number. | 2 |
| **INTRODUCTION** | | |  |
| Rationale | 3 | Describe the rationale for the review in the context of what is already known. | 4 |
| Objectives | 4 | Provide an explicit statement of questions being addressed with reference to participants, interventions, comparisons, outcomes, and study design (PICOS). | 5 |
| **METHODS** | | |  |
| Protocol and registration | 5 | Indicate if a review protocol exists, if and where it can be accessed (e.g., Web address), and, if available, provide registration information including registration number. | 6 |
| Eligibility criteria | 6 | Specify study characteristics (e.g., PICOS, length of follow-up) and report characteristics (e.g., years considered, language, publication status) used as criteria for eligibility, giving rationale. | 6 |
| Information sources | 7 | Describe all information sources (e.g., databases with dates of coverage, contact with study authors to identify additional studies) in the search and date last searched. | 6 |
| Search | 8 | Present full electronic search strategy for at least one database, including any limits used, such that it could be repeated. | 6 and Additional file 2 |
| Study selection | 9 | State the process for selecting studies (i.e., screening, eligibility, included in systematic review, and, if applicable, included in the meta-analysis). | 6-7 |
| Data collection process | 10 | Describe method of data extraction from reports (e.g., piloted forms, independently, in duplicate) and any processes for obtaining and confirming data from investigators. | 7 |
| Data items | 11 | List and define all variables for which data were sought (e.g., PICOS, funding sources) and any assumptions and simplifications made. | 7 |
| Risk of bias in individual studies | 12 | Describe methods used for assessing risk of bias of individual studies (including specification of whether this was done at the study or outcome level), and how this information is to be used in any data synthesis. | 7 |
| Summary measures | 13 | State the principal summary measures (e.g., risk ratio, difference in means). | 7 |
| Synthesis of results | 14 | Describe the methods of handling data and combining results of studies, if done, including measures of consistency (e.g., I^2^) for each meta-analysis. | 7-8 |

| Risk of bias across studies | 15 | Specify any assessment of risk of bias that may affect the cumulative evidence (e.g., publication bias, selective reporting within studies). | 8 |
| --- | --- | --- | --- |
| Additional analyses | 16 | Describe methods of additional analyses (e.g., sensitivity or subgroup analyses, meta-regression), if done, indicating which were pre-specified. | 8-9 |
| **RESULTS** | | |  |
| Study selection | 17 | Give numbers of studies screened, assessed for eligibility, and included in the review, with reasons for exclusions at each stage, ideally with a flow diagram. | 9  Figure 1 |
| Study characteristics | 18 | For each study, present characteristics for which data were extracted (e.g., study size, PICOS, follow-up period) and provide the citations. | 9  Table 1 |
| Risk of bias within studies | 19 | Present data on risk of bias of each study and, if available, any outcome level assessment (see item 12). | 9-10 |
| Results of individual studies | 20 | For all outcomes considered (benefits or harms), present, for each study: (a) simple summary data for each intervention group (b) effect estimates and confidence intervals, ideally with a forest plot. | 9-10 |
| Synthesis of results | 21 | Present results of each meta-analysis done, including confidence intervals and measures of consistency. | 10-11 |
| Risk of bias across studies | 22 | Present results of any assessment of risk of bias across studies (see Item 15). | 10-11 |
| Additional analysis | 23 | Give results of additional analyses, if done (e.g., sensitivity or subgroup analyses, meta-regression [see Item 16]). | 10-11 |
| **DISCUSSION** | | |  |
| Summary of evidence | 24 | Summarize the main findings including the strength of evidence for each main outcome; consider their relevance to key groups (e.g., healthcare providers, users, and policy makers). | 12 |
| Limitations | 25 | Discuss limitations at study and outcome level (e.g., risk of bias), and at review-level (e.g., incomplete retrieval of identified research, reporting bias). | 16 |
| Conclusions | 26 | Provide a general interpretation of the results in the context of other evidence, and implications for future research. | 17 |
| **FUNDING** | | |  |
| Funding | 27 | Describe sources of funding for the systematic review and other support (e.g., supply of data); role of funders for the systematic review. | 19 |

**Appendix file 2: Search Strategy**

Database: PubMed, Embase, Cochrane library, Web of Science, Wanfang, CNKI (Search completed 15th March 2025)

--------------------------------------------------------------------------------------------------------------------------------------------------------------------------------------------------------

**PubMed**

(((((((((Chronic Kidney Insufficiency[Title/Abstract]) OR (Chronic Kidney Insufficiency[Title/Abstract])) OR (Chronic Kidney Insufficiencies[Title/Abstract])) OR (Chronic Renal Insufficiency[Title/Abstract])) OR (Chronic Kidney Diseases[Title/Abstract])) OR (Chronic Kidney Disease[Title/Abstract])) OR (Chronic Renal Diseases[Title/Abstract])) OR (Chronic Renal Disease[Title/Abstract])) OR ("Renal Insufficiency, Chronic"[Mesh])) AND (((((((creatinine/cystatin C ratio[Title/Abstract]) OR (creatinine to cystatin C ratio[Title/Abstract])) OR (creatinine over cystatin C ratio[Title/Abstract])) OR (creatinine-to-cystatin C ratio[Title/Abstract])) OR (sarcopenia index[Title/Abstract]))))

-------------------------------------------------------------------------------------------------------------------------------------------------------------------------------------------------------

**Embase**

#15 #7 AND #14

#14 #9 OR #10 OR #11 OR #12 OR #13

#13 'sarcopenia index':ab,ti AND [embase]/lim

#12 'creatinine-to-cystatin C ratio':ab,ti AND [embase]/lim

#11 'creatinine over cystatin C ratio':ab,ti AND [embase]/lim

#10 'creatinine to cystatin C ratio':ab,ti AND [embase]/lim

#9 'creatinine/cystatin C ratio':ab,ti AND [embase]/lim

#8 #1 OR #2 OR #3 OR #4 OR #5 OR #6 OR #7

#7 'Chronic Kidney Insufficiency':ab,ti AND [embase]/lim

#6 'Chronic Kidney Insufficiencies':ab,ti AND [embase]/lim

#5 'Chronic Kidney Disease':ab,ti AND [embase]/lim

#4 'Chronic Kidney Diseases':ab,ti AND [embase]/lim

#3 'Chronic Renal Disease':ab,ti AND [embase]/lim

#2 'Chronic Renal Diseases':ab,ti AND [embase]/lim

#1 'Chronic Kidney Disease'/exp

------------------------------------------------------------------------------------------------------------------------------------------------------------------------------------------------------

**Cochrane library**

ID Search

#1 MeSH descriptor: [chronic kidney disease] explode all trees

#2 ("chronic kidney disease"):ti,ab,kw (Word variations have been searched)

#3 ("chronic renal disease"):ti,ab,kw (Word variations have been searched)

#4 ("chronic renal diseases"):ti,ab,kw (Word variations have been searched)

#5 ("chronic kidney diseases"):ti,ab,kw (Word variations have been searched)

#6 ("chronic kidney insufficiency"):ti,ab,kw (Word variations have been searched)

#7 ("chronic kidney insufficiencies "):ti,ab,kw (Word variations have been searched)

#8 ("chronic renal insufficiency"):ti,ab,kw (Word variations have been searched)

#9 ("chronic renal insufficiencies"):ti,ab,kw (Word variations have been searched)

#10 #1 OR #2 OR #3 OR #4 OR #5 OR #6 OR #7 OR #8 OR #9

#11 ('sarcopenia index'):ti,ab,kw (Word variations have been searched)

#12 ('creatinine-to-cystatin C ratio'):ti,ab,kw (Word variations have been searched)

#13 ('creatinine over cystatin C ratio'):ti,ab,kw (Word variations have been searched)

#14 ('creatinine to cystatin C ratio'):ti,ab,kw (Word variations have been searched)

#15 ('creatinine/cystatin C ratio*'):ti,ab,kw (Word variations have been searched)

#16 #11 OR #12 OR #13 OR #14 OR #15

#17 #10 AND #16

---------------------------------------------------------------------------------------------------------------------------------------------------------------------------------------------------------

**Web of Science**

1. sarcopenia index [topic]

2. creatinine-to-cystatin C ratio [topic]

3 creatinine over cystatin C ratio [topic]

4. creatinine to cystatin C ratio [topic]

5. creatinine/cystatin C ratio [topic]

6. or/1-5

7. chronic kidney disease [topic]

8. chronic kidney insufficiency [topic]

9. chronic kidney insufficiencies [topic]

10. chronic kidney diseases [topic]

11. chronic renal disease [topic]

12. chronic renal diseases [topic]

13. or/7-12

14. 6 and 13

------------------------------------------------------------------------------------------------------------------------------------------------------------------------------------------------------

Wanfang database

主题：（慢性肾功能不全 or 慢性肾脏疾病 or 慢性肾功能衰竭 or 透析 or 腹膜透析 or 血液透析）and 主题：（肌酐/胱抑素C or 肌肉减少症指数 or 肌酐与胱抑素C比值 or 少肌症指数）

----------------------------------------------------------------------------------------------------------------------------------------------------------------------------------------------------

China National Knowledge Infrastructure database

TKA=(“慢性肾功能不全” + “慢性肾脏疾病” + “慢性肾功能衰竭”+ “血透” + “腹膜透析”+“血透” + “血液透析”）and TKA=(“肌酐/胱抑素C” or “肌肉减少症指数” or “少肌症指数”) **Additional file 3: Data related to the characteristics of patients with CKD**

|  | CKD duration | Definition of sarcopenia/muscle outcomes | eGFR  ml/min/1.73m^2^ | Dialysis vintage, year | Proteinuria | Adjusted variables |
| --- | --- | --- | --- | --- | --- | --- |
| Hyun 2022 | NA | NA | 53.6 | NA | UACR (urea protein-creatinine ratio: 348 mg/g | Age, sex, BMI, DM, HTN, myocardial infarction, cancer, and laboratory variables including hemoglobin, serum albumin, C-reactive protein, uricacid, and Egfrckd - EPICr – Cystatin C |
| Hwang 2022 | NA | NA | 50.6 | NA | Dipstickurineprotein≥1: 45% | Age, sex, history of diabetes, albumin, uric acid, total cholesterol, phosphorus, hemoglobin, and Total Kt/V. |
| Lin 2019 | presence of kidney damage for more than three months | Low muscle strength was defined as an HGS less than 26 kg for men and 18 kg for women, according to the Asian Working Group for Sarcopenia (AWGS) criteria | 36.5 | NA | NA | Age, gender, diabetes, CV disease, WC, body fat mass, albumin, eGFRcys and Cr/CysC, hemoglobin, |
| Lin 2021 | presence of kidney damage for more than three months | the sarcopenia indices were calculated as follows: Cr/CysC = serum Cr (mg/dL)/serum CysC (mg/L) | 32 | NA | UPCR (urea protein-creatinine ratio: 900 mg/g | Age, sex, BMI, DM, CV disease, malignancy, hemoglobin, albumin, TCH, BUN, and eGFRcys. |
| Rizk 2023 | Using ICD-9 and ICD-10 | NA | 62.6 | NA | NA | age, gender, race, ethnicity, myocardial infarction, congestive heart failure, peripheral vascular disease, dementia, cerebrovascular disease, chronic pulmonary disease, rheumatologic disease, pepticulcer disease, liver disease, cancer, diabetes, paralysis, acquired immunodeficiency syndrome, and Charlson comorbidity index |
| Yajima 2023 | NA | Based on the Sarcopenia guidelines of the Asian Working Group (AWGS) 2019, sarcopenia was diagnosed as a combination of reduced muscle strength (women: HGS <18 kg; men: HGS< 28 kg) and decreased muscle mass volume (women: SMI <5.7 kg/m2; men: SMI <7.0 kg/m2). | NA | 4.6 (2.0-7.6) | NA | Age, gender |
| An 2022 | met the blood and urine criteria for CKD | According to the 2019 Asian Working Group for Sarcopenia (AWGS) guidelines, low muscle mass was defined as an LTI of <5.7 kg/m2 for females and <7.0 kg/m2 for males | 40 | NA | NA | Age, BMI, diabetes mellitus, hypertension, albumin, hemoglobin, eGFR. |
| Shi 2023 | NA | NA | 95 | NA | NA | Age, gender, and ethnicity, SBP, LDL-C, BMI, HbA1c, smoking, eGFR |
| Zhang 2025 | anuric for over three months and have undergone Pd for a minimum of three months | NA | NA | NA | NA | Age, sex, history of diabetes, albumin, uric acid, total cholesterol, phosphorus, hemoglobin, hs-CRP, and total Kt/V |

**Additional file 4: Quality assessment and overall risk of bias of included studies**


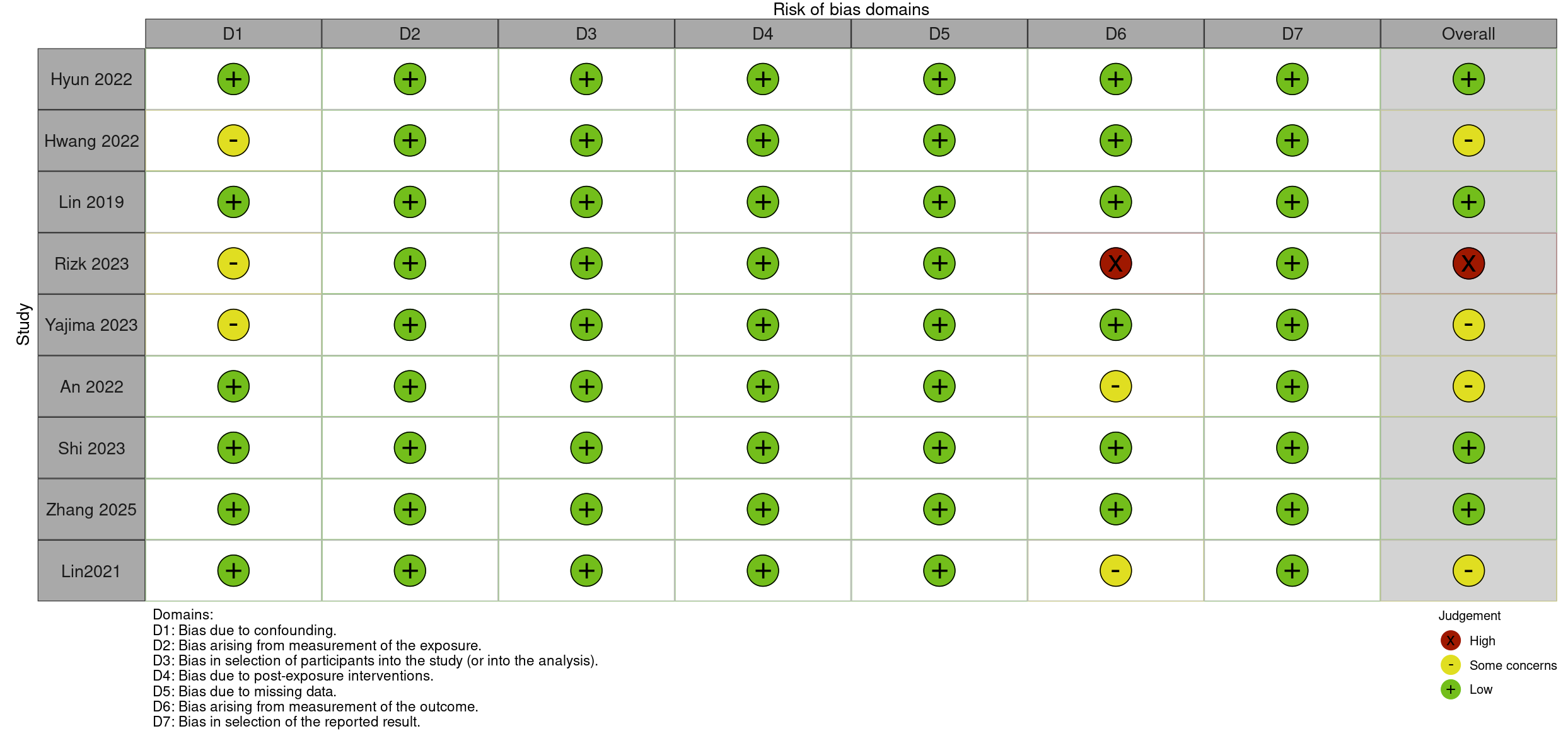


**Additional file 5: GRADE evaluation**


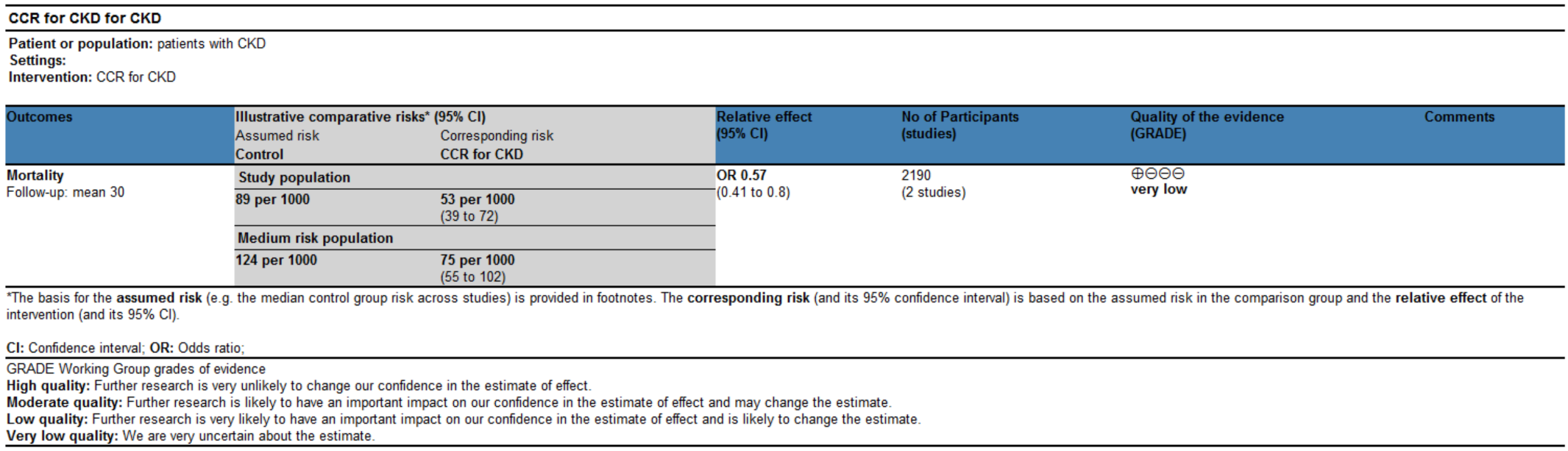


**Additional file 6: Post-hoc meta-regression analyses of the primary outcome**

We conducted post-hoc meta-regression analyses for dialysis status(nd), eGFR, and age. The results of these regressions indicated that these factors did not account for the heterogeneity observed in the combined results.

**Model Results:**

estimate se zval pval ci.lb ci.ub ​

intrcpt 0.0719 0.2138 0.3365 0.7365 -0.3471 0.4910

egfr -0.2981 0.1571 -1.8976 0.0578 -0.6059 0.0098 .

---

Signif. codes: 0 ‘***’ 0.001 ‘**’ 0.01 ‘*’ 0.05 ‘.’ 0.1 ‘ ’ 1

> metareg(A, age)

Mixed-Effects Model (k = 4; tau^2 estimator: DL)

tau^2 (estimated amount of residual heterogeneity): 0.0133 (SE = 0.0418)

tau (square root of estimated tau^2 value): 0.1152

I^2 (residual heterogeneity / unaccounted variability): 32.36%

H^2 (unaccounted variability / sampling variability): 1.48

R^2 (amount of heterogeneity accounted for): 65.84%

Test for Residual Heterogeneity:

QE(df = 2) = 2.9568, p-val = 0.2280

Test of Moderators (coefficient 2):

QM(df = 1) = 2.2344, p-val = 0.1350

**Model Results:**

estimate se zval pval ci.lb ci.ub ​

intrcpt -0.0234 0.2135 -0.1098 0.9126 -0.4420 0.3951

age -0.1801 0.1205 -1.4948 0.1350 -0.4163 0.0561

---

Signif. codes: 0 ‘***’ 0.001 ‘**’ 0.01 ‘*’ 0.05 ‘.’ 0.1 ‘ ’ 1

> metareg(A, nd)

Mixed-Effects Model (k = 4; tau^2 estimator: DL)

tau^2 (estimated amount of residual heterogeneity): 0.0329 (SE = 0.0495)

tau (square root of estimated tau^2 value): 0.1813

I^2 (residual heterogeneity / unaccounted variability): 70.91%

H^2 (unaccounted variability / sampling variability): 3.44

R^2 (amount of heterogeneity accounted for): 15.38%

Test for Residual Heterogeneity:

QE(df = 2) = 6.8743, p-val = 0.0322

Test of Moderators (coefficient 2):

QM(df = 1) = 1.1226, p-val = 0.2894

**Model Results:**

estimate se zval pval ci.lb ci.ub ​

intrcpt -1.2150 0.8193 -1.4829 0.1381 -2.8209 0.3908

nd 0.4493 0.4240 1.0595 0.2894 -0.3818 1.2804

---

Signif. codes: 0 ‘***’ 0.001 ‘**’ 0.01 ‘*’ 0.05 ‘.’ 0.1 ‘ ’ 1
